# *CPEB1* deletion is not a common explanation for premature ovarian insufficiency in a Chinese cohort

**DOI:** 10.1186/s13048-020-00630-x

**Published:** 2020-04-30

**Authors:** Wenlin Jiao, Shidou Zhao, Ran Liu, Ting Guo, Yingying Qin

**Affiliations:** 1 Center for Reproductive Medicine, Cheeloo College of Medicine, Shandong University, Jinan, 250012 Shandong China; 2 National Research Center for Assisted Reproductive Technology and Reproductive Genetics, Shandong University, Jinan, 250012 Shandong China; 3 Key laboratory of Reproductive Endocrinology of Ministry of Education, Shandong University, Jinan, 250012 Shandong China; 4 Shandong Provincial Clinical Medicine Research Center for Reproductive Health, Shandong University, Jinan, 250012 Shandong China

**Keywords:** *CPEB1*, POI, Microdeletion, Meiosis, Polyadenylation

## Abstract

**Purpose:**

Premature ovarian insufficiency (POI), which is characterized by early menopause before the age of 40 years, affects approximately 1–5% of women. Cytoplasmic polyadenylation element binding protein 1 (CPEB1) is a post-transcriptional regulatory protein that is highly expressed in germ cells and promotes oocytes maturation, and several studies have found microdeletions of chromosome 15q25.2, which contains the *CPEB1* gene, in POI patients. However, the deleted region also includes other plausible genes, and thus the contribution of *CPEB1* to POI is uncertain. The present study aimed to determine the relationship between *CPEB1* deletion and POI in a Chinese cohort.

**Material and methods:**

Quantitative real-time polymerase chain reaction (qPCR) with primers for exon 4 and exon 11 of *CPEB1* was performed to detect the *CPEB1* deletion in 323 patients with POI and in 300 healthy controls. Subsequent qPCR with primers for each exon of *CPEB1* was performed to precisely localize the deletion locus.

**Results:**

One patient with primary amenorrhea was found to carry a heterozygous deletion of exons 8–12 of the *CPEB1* gene.

**Conclusion:**

Our study is the first to search for *CPEB1* deletions in POI patients using a simple qPCR method, and we show that *CPEB1* deletion is not a common cause for POI in a Chinese cohort.

## Introduction

 Premature ovarian insufficiency (POI) is characterized by cessation of menstruation before 40 years of age combined with a serum level of follicle stimulating hormone (FSH) above 25 IU/L [1]. Approximately 1–5% of women under 40 years old are affected by POI and are at high risk of osteoporosis, cardiovascular disease, and other long-term health complications due to estrogen deficiency, thus adversely affecting women’s health both physiologically and psychologically [2].

The etiology of POI is heterogeneous, including chromosome abnormalities, gene mutations, autoimmune diseases, and iatrogenic factors; however, most cases are idiopathic. Genetic disorders account for 20–25% of POI cases, and chromosomal abnormalities explain 10–15% [3]. Mutations have been identified in the genes participating in meiosis, folliculogenesis, and steroid hormone synthesis, such as *STAG3*, *SYCE1*, *MSH5*, *GDF9*, *NOBOX*, and *FSHR*. However, only a minority of the mutations have been verified through functional assays [3, 4].

The genes involved in chromosome synapsis and homologous recombination during meiosis I are essential for oogenesis and for the maintenance of ovarian function. Deficiencies in the proteins encoded by these genes lead to oocyte apoptosis and infertility. Cytoplasmic polyadenylation element binding protein 1 (CPEB1) has been found to regulate the translation of essential genes – such as *SYCP1* and *SYCP3 –* that are responsible for synaptonemal complex formation and homologous chromosome recombination [5]. *Cpeb1-*knockout mice show atrophic ovaries devoid of oocytes, mimicking the phenotype of human POI [6]. Previous studies found that *CPEB1* heterozygous deletions occur in 0.33–2.9% of POI patients of Caucasian descent using array comparative genomic hybridization assay (CGH array) or quantitative multiplex PCR of short fluorescent fragments techniques (Table 1). Here, we used quantitative real-time polymerase chain reaction (qPCR) to locate the precise deletion region of *CPEB1* in 323 Chinese patients with sporadic POI.

**Table 1:** Previous studies of *CPEB1* deletions in POI patients

Case No.	Deletion	Type of amenorrhea	Familial history	Other features	Prevalence	Citation
Case 1	15q25.2	Secondary	–	–	0.33%	Tsuiko et al.
Case 2	15q25.2	Primary	–	Behavioral disorders, progressive intellectual deficiency	1.15%	Hyon et al.
Case 3	15q25.2	Secondary	–	–		
Case 4	15q25.2	Primary	Yes	–		
Case 5	15q25.2	Primary	–	–	1.12%	McGuire et al.
Case 6	15q25.2	Primary	Yes	–	2.9%	Bestetti et al.
Case 7	15q25.2	Primary	Yes	–		

## Materials and methods

### Participant population

The inclusion criteria for POI were cessation of menstruation before 40 years of age and at least twice serum follicle stimulating hormone (FSH) concentrations exceeding 25 IU/L. In the present study, 123 patients with primary amenorrhea and 200 patients with secondary amenorrhea were recruited from Shandong Provincial Hospital Affiliated to Shandong University. Women with a history of pelvic surgery, chemotherapy, radiation therapy, or chromosome abnormalities were excluded. Three hundred women above 40 years of age with normal menorrhea and delivery history were recruited as controls. Written informed consents were obtained from all participants. The study was approved by the Institutional Review Board of Center for Reproductive Medicine, Shandong University.

### Quantitative real-time polymerase chain reaction

Genomic DNA was extracted from the peripheral blood of all participants. The *CPEB1* gene includes 12 exons, and qPCR with primers recognizing sequences in exon 4 and exon 11 was performed to screen for the gene deletion according to a previous CGH array with probes targeting exon 4 of *CPEB1*.

The 114 bp fragment targeting the *ACTB* gene was used as the normalization control (Table 2). DNA samples from women with no microdeletions according to the CGH array were used as the negative control in each assay. To determine the gene dosage of every amplified region, dosage quotients were obtained by dividing the ratio of the sample (2^-∆∆CT^ of the *CPEB1* fragment compared to *ACTB*) by the corresponding ratio of the negative control DNA. This equation provided a theoretical dosage quotient value of 1.0 for two copies and a value of 0.5 for deletions. Three independent replicates were performed for each case, and independent samples t-tests were used to test for differences. We identified a *CPEB1* deletion in one patient, and qPCR with primers for each exon of *CPEB1* was performed to localize the deletion region.

**Table 2:** Primers for qPCR of *CPEB1* and *ACTB*

	Forward primer	Reverse primer
Exon 1	5′-AGCGGCTCGTAGGAGCTTCAT-3’	5′-TTACCAGCGGGAACGCCAT-3’
Exon 2	5′-GATAAAAGATTGCTGGGACAACC-3’	5′-CAGATGCCTACCACGTTCAAGT-3’
Exon 3	5′-GCTTTTCCCAACCTCTGCG-3’	5′-TACCCCAGCCAACTCATTCTC-3’
Exon 4	5′-AGTTTCCAGCACCCTCAGTTAG-3’	5′-CACAATAATCTCCACTCCTCCC-3’
Exon 5	5′-CTTCGCATTTCTCCACCTCTG-3’	5′-TGGTTGGGGAGGGAGTGACT- 3’
Exon 6	5′-AAGCCACCTGTACCTGGAGTG-3’	5′-GCCCCACCCTTCAACTCTTA-3’
Exon 7	5′-CTTCTGCCATTCTTTTCTGTCTC-3’	5′-GTGCCCACCATGTTACCAAC-3’
Exon 8	5′-CTGTCCGATCCTTGCTTCA-3’	5′-CCTGCCTATCCACCTACCAC-3’
Exon 9	5′-AGGGCGTTAGCTTAGCTTCAG-3’	5′-GGCATACACCACTCCACCAA-3’
Exon 10	5′-CCAACGGAGTTACCTGAAAGC-3’	5′-CGTACAAAGACCAAGCCCAC-3’
Exon 11	5′-TGAATCCCAGAGGCATCCAG-3’	5′-ACAGAAGAAAGGACCAGGCT-3’
Exon 12	5′-TGTAACAAGGATGGTGGGTTTG-3’	5′-GTTTGGAGAAGGGTGGGAGAC-3’
ACTB	5′-ATTCCTATGTGGGCGACGA-3’	5′-TGTGGTGCCAGATTTTCTCC-3’

## Results

**Fig. 1:**
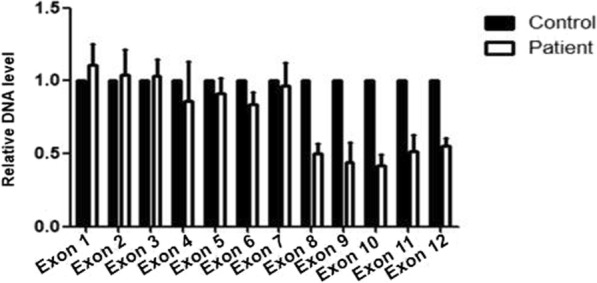
Heterozygous deletion of *CPEB1* exons 8–12 was identified in one POI patient

In our qPCR of *CPEB1* exon 4 and exon 11 in 323 patients and 300 controls, we found that the DNA dosage of exon 11 was reduced by half in one patient. Further qPCR for each exon of *CPEB1* identified a heterozygous deletion of exons 8–12(Fig. 1).

The patient carrying the *CPEB1* deletion was diagnosed with POI at 18 years of age presenting with primary amenorrhea and elevated FSH level. Ultrasound examination showed small ovaries and no visible follicles.

## Discussion

Our qPCR experiment in 323 cases with sporadic POI provides the first precise identification of a *CPEB1* microdeletion in a Chinese population, supporting the existence of *CPEB1* haploinsufficiency consisted in POI pathogenesis, albeit with a low frequency of 0.31%.

CPEB1 is an important post-transcriptional regulatory protein that binds to the CPE region of target mRNAs and promotes the polyadenylation and translation of the mRNAs [7, 8]. *CPEB1* is preferentially expressed in germ cells and regulates the translation of crucial genes involved in meiosis and oogenesis, such as the synapsis complex protein genes *SYCP1* and *SYCP3*. Oocytes that are deficient in CPEB1 arrest at the pachytene stage due to abnormal synaptonemal complex formation, resulting in ovarian atresia and infertility [6]. Moreover, in *Cpeb1-*knockdown oocytes, the expression of growth differentiation factor 9 (GDF9), which is required for folliculogenesis, is significantly reduced due to a shortened mRNA poly(A) tail, indicating a pivotal role for *CPEB1* in oogenesis after birth [9]. Our results combined with previous studies support the hypothesis that *CPEB1* deficiency in humans might accelerate follicle atresia due to disrupted meiosis and oogenesis, thus resulting in ovarian failure at an earlier age, as is the case in POI.

In previous studies using CGH array or SNP array analysis, a total of seven POI cases were found to carry a heterozygous deletion of chromosome 15q25.2, where *CPEB1* is partially localized (Table 1). Hyon et al. [10] reported three patients carrying the 15q25.2 deletion in 259 (1.15%) POI cases, and two of them presented with primary amenorrhea and the other presented with secondary amenorrhea. McGuire et al. [11] screened 89 POI patients and identified the 15q25.2 deletion in one patient (1.12%) suffering from primary amenorrhea. Tsuiko et al. [12] observed one case with the 15q25.2 deletion in the cohort samples from a bio bank (1/301, 0.33%). Finally, the study by Bestetti et al. [13] used a high-resolution CGH-array method and found 2 patients among 67 POI cases (2.9%) harboring the deletion. However, the deleted region of 15q25.2 contains more than 10 genes, and whether the *CPEB1* deletion was completely or partially responsible for POI remained uncertain. Moreover, the probes used previously to target the *CPEB1* gene were localized to exons 1–5, which could not identify any deficiencies in other exons. In addition, the breakpoint of the deletion was ambiguous. Therefore, the causative role of *CPEB1* deletion in the pathogenesis of POI required more evidence. In the present study, two pairs of qPCR primers recognizing sequences in *CPEB1* exon 4 and exon 11 were designed, which expanded the probe-targeting region. Similarly to previous studies, the frequency of *CPEB1* microdeletion (1/323, 0.31%) in our cohort was low, indicating that *CPEB1* microdeletion is not a common cause for POI in a Chinese population. Furthermore, we identified the specific deletion region of *CPEB1* as being exons 8–12, which indicates the vital role of this region for the function of CPEB1.

**Fig. 2:**
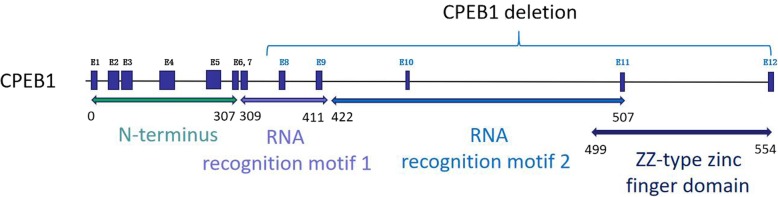
The gene structure of *CPEB1* and the deletion region identified in the present study

The CPEB1 protein is composed of three regions – an amino-terminal portion with no obvious functional motifs, two RNA recognition motifs (RRMs), and a zinc finger motif [14]. Previous studies have demonstrated that the RRMs and the zinc finger motifs are essential for the RNA binding capacity of CPEB1 [15], and Merkel et al. [16] found that the C-Terminal region of CPEB1 has the potential to recruit other proteins during the assembly of the ribonucleoprotein complex. Because the RRMs, zinc finger motifs, and C-terminal region are all encoded by exons 8–12, it can be assumed that the CPEB1 protein translated from the allele without these exons is non-functional (Fig. 2), and thus haploinsufficiency of CPEB1 due to the heterozygous microdeletion is likely to be the causative factor for the POI phenotype in a small subset of patients.

The present study has some limitations that must be noted. The primary qPCR only tested exon 4 and exon 11 of *CPEB1*, which could have missed microdeletions outside this region. Moreover, the functional study was not performed given the fact that the proteins regulated by CPEB1 are expressed in oocytes during meiosis or folliculogenesis, which cannot be cultured in vitro. Considering the limitation, animal models with *CPEB1* deletion in exons 8–12 might be generated to further illustrate the pathogenic mechanism of such deficiency on mammalian oogenesis and ovarian function.

## Conclusion

Taken together, our results further illustrate that deficiency in *CPEB1*, which is required for synaptonemal complex formation and oogenesis, contributes to only a small minority of POI cases.
